# A Fast Neighbor Discovery Algorithm in WSNs†

**DOI:** 10.3390/s18103319

**Published:** 2018-10-03

**Authors:** Liangxiong Wei, Weijie Sun, Haixiang Chen, Ping Yuan, Feng Yin, Qian Luo, Yanru Chen, Liangyin Chen

**Affiliations:** 1 College of Computer Science, Sichuan University, Chengdu 610065, China; weilx_scu@aliyun.com (L.W.); xiaowei201102@163.com (W.S.); scuchx15@163.com (H.C.); 2 School of Mathematics and Information Engineerging, Chongqing University of Education, Chongqing 400065, China; pypingyuan@163.com; 3 School of Computer Science and Technology, Southwest University for Nationalities, Chengdu 610225, China; yf_eagle@aliyun.com; 4 Second Research Institute, General Administration of Civil Aviation of China, Chengdu 610041, China; caacsri_luoqian@163.com; 5 Institute for Industrial Research, Sichuan University, Chengdu 610065, China

**Keywords:** neighbor discovery, WSNs, energy efficiency

## Abstract

With the quick development of Internet of Things (IoT), one of its important supporting technologies, i.e., wireless sensor networks (WSNs), gets much more attention. Neighbor discovery is an indispensable procedure in WSNs. The existing deterministic neighbor discovery algorithms in WSNs ensure that successful discovery can be obtained within a given period of time, but the average discovery delay is long. It is difficult to meet the need for rapid discovery in mobile low duty cycle environments. In addition, with the rapid development of IoT, the node densities of many WSNs greatly increase. In such scenarios, existing neighbor discovery methods fail to satisfy the requirement in terms of discovery latency under the condition of the same energy consumption. This paper proposes a group-based fast neighbor discovery algorithm (GBFA) to address the issues. By carrying neighbor information in beacon packet, the node knows in advance some potential neighbors. It selects more energy efficient potential neighbors and proactively makes nodes wake up to verify whether these potential neighbors are true neighbors, thereby speeding up neighbor discovery, improving energy utilization efficiency and decreasing network communication load. The evaluation results indicate that, compared with other methods, GBFA decreases the average discovery latency up to 10.58% at the same energy budget.

## 1. Introduction

With the quick development of Internet of Things (IoT), one of its important supporting technologies, i.e., wireless sensor networks (WSNs), gets more attention [1]. WSN is self-organizing network which contains lots of sensors with very limited energy. Sensed data are forwarded in a multi-hop manner to sink node or base station. WSNs are indispensable for IoT, especially in the cases where sensed data volume becomes more huge with the development of IoT. These data can be processed in sink node firstly and only a fraction of data is sent to Cloud, hence the network communication load can be reduced significantly. In addition, in the cases where a large number of devices want to access internet [2], WSN-based manner has much larger connection capacity, compared with direct connection way. WSN-based applications in agriculture [3], environment [4], industrial [5], biomedicine, military and other fields [6], will increase rapidly with the evolution of IoT.

As a prerequisite for networking and routing, neighbor discovery is a key procedure [7]. Neighbor discovery is indispensable not only in the initial phase but also at any time when the network is beginning to work, since new sensors may be placed [8,9]. In addition, the neighbors of nodes change quickly in mobile WSNs. Neighbor discovery operation needs work continually in the entire lifetime of the network [10].

Due to limited power of sensor nodes in WSNs [6,11], nodes usually work with low duty cycle (LDC) mode to prolong the service life. It means that the node is awake in several slots and asleep during the remaining slots. In LDC mode, for two nodes that lie in the communication range of each other, it is difficult to make them be active at the same time. Since the energy is mainly consumed by active slots, we use the duty cycle (for short, DC, which is equal to the active slot number divided by the total slots) as the indicator of energy consumption, like many existing methods [7,12]. The energy consumption of one active slot is the same as other active slots and contains the energy consumed in computing, communicating, etc. In the calculating of DC value, the energy consumption of one active slot and one sleep slot is regarded as 1 and 0 unit, respectively. If the energy consumptions of active slots are different, i.e., unequal slot size, we can give different weighted values to them. In general, lower DC and lower discovery latency both are required by neighbor discovery methods. The smaller both the energy consumption (or DC) and discovery latency are, the more energy efficient the algorithm is. To compare the energy efficiency of different methods, we can make the DCs of different methods unchanged and observe the changes of discovery latencies, or the discovery latencies of different methods can be equal and observe the changes of DCs. We introduce the concept of energy efficiency to fairly and conveniently compare the performances of all related methods. Many neighbor discovery algorithms have been proposed to improve the energy efficiency. The existing algorithms are divided into probabilistic algorithms and deterministic ones. In probabilistic neighbor discovery algorithms, the state of node, i.e., active or asleep state, follows a probabilistic distribution of time. These kinds of algorithms can get a shorter average discovery delay but can’t guarantee the detection of a neighbor within a given latency. In the deterministic discovery algorithm, the node adopts the predetermined wake-up schedule to ensure that all the neighbors are discovered within the given delay, but the average discovery latency is often longer.

In recent years, more and more researchers have presented some neighbor discovery middleware, which use transitivity of neighbor relations to speed up neighbor discovery by sharing neighbor information, such as WiFlock [13], Acc [14], Group-based [10] and EQS (Extended Quorum Systems) [9]. In the WiFlock algorithm [13], all nodes in the range of one-hop are regarded as one group. By uniformly adjusting distribution of the nodes’ active slots in the group, the nodes outside the group can be found more quickly, thereby speeding up the neighbor discovery process. In the Acc [14] algorithm, the node selects some additional slots to be active. Each slot owns a slot gain value at current slot, and the slot gain is decided by temporal diversity and spatial similarity. The slot gains of selected slots must be larger than the gains of other slots. The main limitation of Acc is that the calculation of temporal diversity requires information about many neighbors. Otherwise, it will cause the additional active slots to be noneffective. As a result, Acc performs poorly when node density in the network is low. The EQS method [9] uses quorum graph theory to remove the unnecessary working slots to improve energy efficiency. The Group-based algorithm [10] adds a small amount of additional active slots, used to recommend and verify the neighbor relationship, which greatly speeds up neighbor discovery. However, Group-based is not very efficient in the selection of verification nodes. There is still room for further improvement in energy usage efficiency. Overall, these methods can be divided into two categories in accordance with adding efficient active slots, like Acc and Group-based, or removing ineffective active slots, like EQS. There are two ideas of how to add efficient active slots, namely mining of wake-up patterns (like Acc) and collaborative neighbor discovery (like Group-based). Group-based methods, included our proposed method, are complementary with other kinds of discovery middleware rather than conflicting with them. It means that two or more kinds of middleware can be combined to work. For example, both EQS and Group-based (can be replaced by our proposed method) can be employed to remove ineffective active slots and add efficient active slots, respectively. In addition, when node density is low, Group-based or our proposed method can be used. However, when node density is high, both Acc and Group-based (or our proposed method) method can be employed.

With the rapid development of IoT, the node density of many WSNs, i.e., the node number in unit area, greatly increases. In these scenarios, existing neighbor discovery methods fail to satisfy the requirements in terms of energy efficiency, communication traffic load, etc. To address this issue, this paper proposes a group-based fast neighbor discovery, i.e., GBFA (Group-based Fast Algorithm), based on the existing Group-based method [10], to highly efficiently speed up the discovery process. The key to our method is selecting an energy efficient slot to be awake to reduce discovery latency and decrease communication traffic load under the conditions of a given energy budget. Therefore, it is very suitable for IoT-oriented WSN scenarios. Specifically, for one sender, its beacon packets contain its neighbor information. When another node receives the beacon packet, the receiver will know the wake-up schedules of the sender’s neighbors. The neighborship between the receiver and sender’s neighbors will be verified when the sender’s neighbors wake up the next time. In GBFA, low efficient verifications are filtered out as much as possible by two ways. Our main contributions are as follows:

- The node can broadcast its neighbor information. The node that receives the neighbor information can verify early whether the receiver and sender’s neighbors are neighbors. We quantify the contribution of each verification to early discovery time (early discovery time refers to the discovery time if only a pairwise method is used subtracts the discovery time when a discovery middleware, i.e., GBFA, is also employed) and filter out the unnecessary verifications that have less contribution degree.
- Carrying neighbor information in a beacon packet makes some neighbor discoveries much earlier, but the length of beacon packet increases and hence adds the possibility of packet collisions, which decreases performance of the network. In this paper, we tailor a packet format with multiple frames for each beacon packet. As a result, GBFA can continue working when the packet collision number is controlled below a threshold value.
- We evaluated and compared GBFA with the existing algorithms. The results show that our method outperforms others in various cases.

The rest of the paper is structured as follows. In Section 2, main previous neighbor discovery algorithms in WSNs are briefly introduced. Section 3 presents the motivation of GBFA. In Section 4, our proposed method is described in detail. In addition, in Section 5, simulations are designed to evaluate our algorithm and we compare it with other methods. Finally, Section 6 and Section 7 discuss the disadvantages of the proposed approach, give our research directions in the future and conclude the paper.

## 2. Related Work

In the early development of WSNs, sensor nodes mainly work in the way of always being active since the application environment is relatively single. During this period, there are some neighbor discovery algorithms that are suitable for this situation, such as [15,16,17]. These algorithms require nodes to be active all the time and are not suitable for LDC WSNs. To meet the demand of LDC, some researchers have proposed some synchronous algorithms based on clock synchronization, such as S-MAC [18]. Although the synchronous algorithms are simple, they need to consume a lot of energy for clock synchronization. It is difficult to synchronize the nodes without knowing other nodes in advance.

Many asynchronous neighbor discovery algorithms have emerged in recent years. Based on whether the discovery latency is finite or not, existing pairwise asynchronous protocols are divided into the probabilistic algorithms [12,19,20] and deterministic ones [8,21,22,23,24,25,26,27,28].

The probabilistic algorithms include Birthday [19], PSBA [20], Panda [12] and so on. In Birthday, each node selects one work state from active and sleep states in each slot with a probability. PSBA leverages on the advantage of both probabilistic and deterministic algorithms, so that the long latency tail is reduced. A latest probabilistic protocol is Panda [12]. In Panda, each sensor remains asleep in an initial step. The sleep time of the node follows an exponential distribution. Following sleep step, sensors wake up and listen for a constant time. If no packet is received in the listen state, the node broadcasts one packet to others.

The earliest deterministic neighbor discovery protocols are quorum-based ones [21,22,29], where time is divided into $m^{2}$ slots. These slots can be regarded as a two-dimensional array. Node can randomly select 1 row and 1 column to be active. This pattern ensures that the overlap of active slots between two nodes is within $m^{2}$ slots. Another kind of earlier protocols is prime-based ones, like Disco [23] and U-connect [24]. Although quorum-based and prime-based methods obtain a small worst bound, they are worse than the probabilistic protocol in the average case. In response to that, Searchlight [7] is proposed, which employs two active slots, i.e., anchor slot (A slot) and probe slot (P slot) in one period. An A slot is fixed in the first slot in each period. Since the length of any period is the same, the offset between the A slots of any two nodes remains unchanged. Then, P slot is introduced to probe the A slot of the other node. The later researchers find that employing unequal-sized slots can obtain good discovery performance. Searchlight-Striped [7], Non-integer [25] and Lightning [26] are included. There are other related methods, i.e., block-based methods [8,27] and Nihao [28]. In block-based methods, block theory is used to obtain a wakeup schedule. Nihao defines dedicated listening and transmitting slots. In each listen slot, the radio remains listening during the whole slot. In each transmitting slot, one beacon is sent at the beginning; then, the node goes back to sleep.

In recent years, more and more researchers have proposed some neighbor discovery middleware that use the transitivity of neighbor relationships to accelerate neighbor discovery by sharing neighbor information. In the WiFlock algorithm [13], all nodes in the range of one-hop neighbors are regarded as one group. By uniformly adjusting distribution of the nodes’ active slots in the group, the nodes outside the group can be found more quickly, thereby speeding up the neighbor discovery process. In the Acc [14] algorithm, each slot has a slot gain value, decided by temporal diversity and spatial similarity. The temporal diversity between a pair of devices is determined by the difference in active slot schedules between them. The spatial similarity between a pair of devices is determined by the spatial closeness between them. Within a given time interval, a small amount of additional slots with optimal slot gains are selected to be active, except for original active slots. The main limitation of Acc is that the calculation of temporal diversity requires information about many neighbors. Otherwise, it will cause the additional acitve slots to be noneffective. As a result, Acc performs poorly when node density in the network is low. The EQS method [9] uses quorum graph theory to remove the unnecessary active slots to improve energy efficiency. The Group-based algorithm [10] adds a small amount of additional wake-up slots, used to recommend and verify neighbor relationships, which greatly speeds up neighbor discovery. However, Group-based is not very efficienct in the selection of verification nodes. There is still room for further improvement in energy usage efficiency. Overall, these methods can be divided into two categories in accordance with adding efficient active slots, like Acc and Group-based, or removing ineffective active slots, like EQS. There are two core ideas of how to add efficient active slots, i.e., mining of wake-up patterns (like Acc) and collaborative neighbor discovery (like Group-based). In this paper, a fast neighbor discovery based on Group-based is proposed to further improve the energy efficiency. Note that our method is complementary with other kinds of discovery middleware rather than exclusive. It means that two or more kinds of middleware can be combined to work. For example, both EQS and GBFA can be employed to remove ineffective active slots and add efficient active slots, respectively. In addition, when node density is low, our proposed method can be used. However, when node density is high, both Acc and GBFA can be employed.

In the end of the section, the main related works are summarized in Table 1.

## 3. Motivation

With the rapid development of IoT, the node densities of many WSNs, especially some industrial WSNs, greatly increase. For example, shipping in the industrial IoT is a complex use case. Thousands of sensors are on dozens of pallets, which is a very dense wireless environment [30], where there are many packet collisions and these collisions cause the great increase of discovery latency. Another example is presented below. Please consider a manufacturing shop where there exists a large number of sensors. Mobile sensors, i.e., the sensors carried by industrial robots, and static sensors coexist. In the complex scenarios, it is extremely challenging to achieve real-time and reliable communication between sensors under the condition of given energy budget. Neighbor discovery in such scenarios also needs high performance in terms of real-time and reliability. In these scenarios, including the two ones stated above, existing neighbor discovery methods fail to satisfy the requirement.

Whether the RF signal of other node can be received or not is the only judgment of neighbor discovery if only pairwise discovery method is used. However, the neighbor table of one node can be used to efficiently find more neighbors. The higher the node density is in WSNs, the more information about neighbor tables can be used. As a result, more energy efficiency improvement of neighbor discovery can be achieved. However, there is one problem that can’t be ignored. For any two sensors A and B, if A receives the neighbor table of B, A will proactively wake up when B’s neighbor wakes up to verify whether they are neighbors. More information about neighbor tables means more proactive active slots. These active slots causes much energy consumption and packet collisions. Thus, it is crucial to carefully select efficient verifications and filter out unnecessary proactive active slots. In the paper, we achieve the goal through two ways. One is estimating the distance between two nodes based on the number of their common neighbors. Another one is selecting the recommended nodes if the early discovery time is more than a given value.

Since efficient verifications greatly reduce early discovery time, GBFA can work with a much lower duty cycle mode in dense IoT-oriented WSNs, compared with other existing methods. Packet collisions have been reduced, real-time and reliable neighbor discovery can be obtained under the condition of given energy budget.

## 4. Method Design

In this section, we firstly give the system assumptions. Next, we give the core idea of our method. To make this easier to follow, an example about the discovery process of GBFA is presented. Finally, two methods of carefully selecting efficient verifications are detailed.

### 4.1. System Assumptions

We make some assumptions for the nodes in WSNs as follows:

- For easy analysis, we first assume that nodes are uniformly deployed in a two-dimensional area. Based on the assumption, we deduce that the distance between two nodes is approximately proportional to the number of their common neighbors in Section 4.3. To extend GBFA to other WSNs, i.e., randomly deployed WSNs, we will study the relationship between the distance and the common neighbor number in this case in the future.
- Each node owns one and only identification (ID) to distinguish the node from others.
- The signal coverage area of any node is a fixed circle.
- The transmitted power and signal frequencies of all nodes are the same, respectively.
- Each node works in a slot-based and LDC way. We ignore the time and energy consumption of transient state, i.e., power-up and power-down state.
- All nodes employ an identical neighbor discovery method.

### 4.2. Core Idea of GBFA

**Figure 1:**
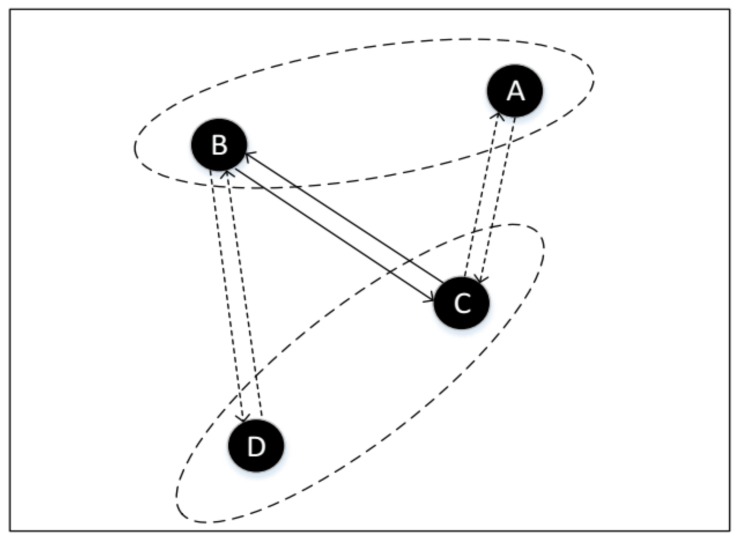
The discovery process of our method.

Our method can be regarded as a neighbor discovery middleware. In an underlying layer, any pairwise neighbor discovery method can be used. GBFA efficiently schedules a small amount of additional active slots to accelerate neighbor discovery process. For any four nodes (see Figure 1, the four black circles denote node A, node B, node C and node D respectively), assume that the neighbor table of B contains A, and the neighbor table of C contains D. In other words, A and B have discovered each other, C and D also have discovered each other through pairwise neighbor discovery. Next, GBFA will work. Specifically, when B and C discover each other, they will send their neighbor information to each other. Therefore, B and C will know the wake-up schedule of D and A, respectively. Since B and C recommend their neighbor information to each other, B and C are called recommenders. A and D are included in the neighbor information of C, B, respectively, and will be recommended in the next successful discovery time. A and D are called recommended nodes. If any node, i.e., B, sends its neighbor information to another, i.e., C,C is called accepted node of B. Because we can’t make sure whether one recommended node is the neighbor of its accepted node, it is necessary to verify whether they are neighbors or not. Since C knows the wake-up schedule of A after recommending, C will proactively wake up when A wakes up the next time to receive a beacon packet of A. B also can verify whether D is its neighbor in the same way.

**Figure 2:**
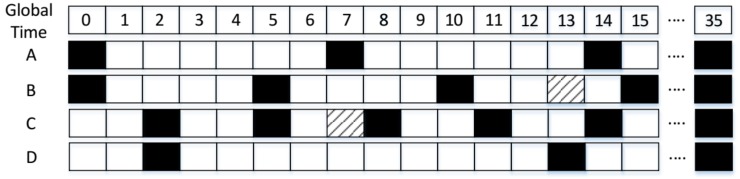
An example of GBFA.

Next, we give an example to describe the process. As Figure 2 shows, A, B, C, and D denote four nodes, any of which can receive the RF signals from the others. The four nodes employ a Disco pairwise method in the underlying layer. Their duty cycles are: $DC_{A}=\frac{1}{7}$, $DC_{B}=\frac{1}{5}$, $DC_{C}=\frac{1}{3}$ and $DC_{D}=\frac{1}{11}$, respectively. Firstly, both A and B wake up in slot 0 and discover each other. Next, C and D discover each other in slot 2. Then, B and C discover each other in slot 5; meanwhile, B recommends its neighbor information (including A’s) to C. As a result, C knows the wake-up schedule of A and wakes up in A’s next active slot, namely slot 7, to verify whether A is its neighbor. Since C can receive the beacon packet of A, C has confirmed that A is its neighbor. Thus, A and C achieve successful neighbor discovery in advance. However, if only the pairwise method is used, the successful discovery between A and C is obtained in slot 14, which is seven slots behind the time when GBFA is used. Similarly, C will recommend its neighbor information (including node D’s) to B at slot 5. B and D can achieve successful neighbor discovery in advance at slot 13, which is 22 slots faster than the time when only the Disco method works.

### 4.3. Selection of Verification Node

From Section 4.2, we can see that an accepted node is required to verify its recommended nodes, since there is no information to confirm that recommended nodes are the neighbors of the accepted node before verification. When accepted nodes and recommended nodes are far apart from each other, there is no probability of becoming neighbors. The advantage of verification in this case disappears. Therefore, it is necessary to carefully select appropriate recommended nodes that have much to contribute to the early discovery time. Next, we will select appropriate verification node by two ways. One is estimating the distance between two nodes based on the number of their common neighbors. Another one is selecting the recommended node if the early discovery time when GBFA is used is more than a threshold value.

Next, we discuss how to estimate the distance between two nodes and how to select verification based on the estimated distance.

**Figure 3:**
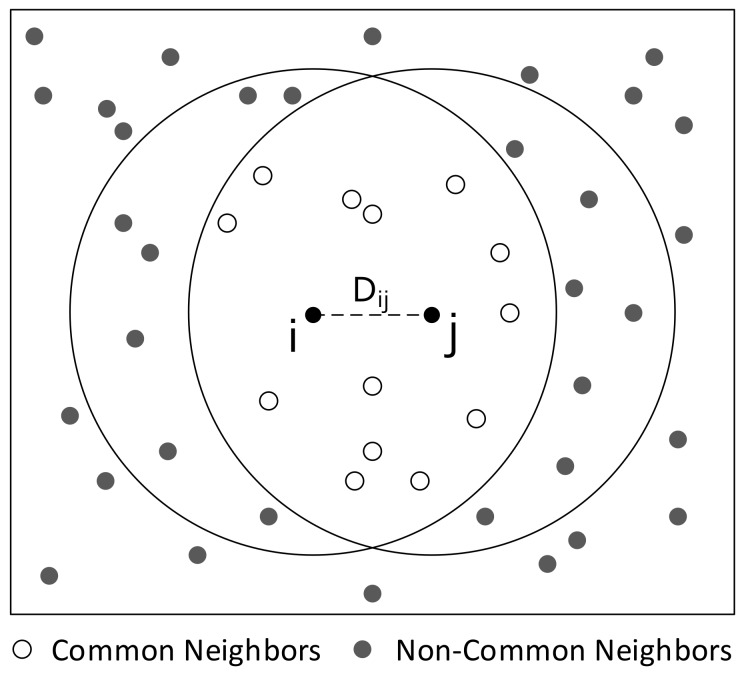
Node uniform distribution.

**Figure 4:**
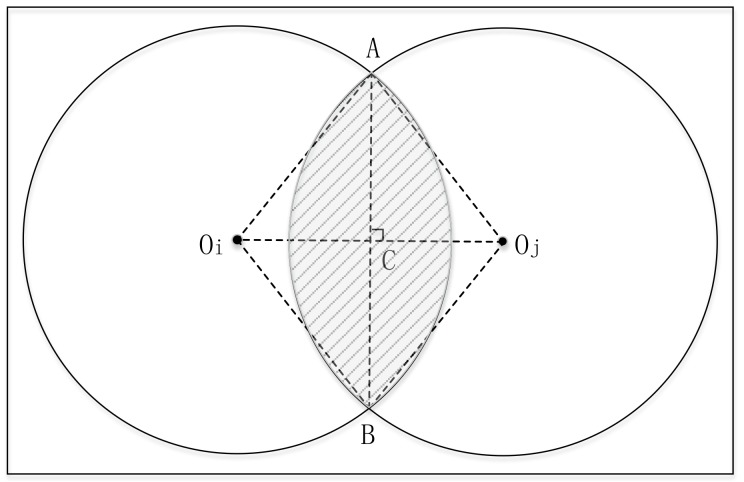
The calculation of overlap area.

For two nodes *i* and *j* (A and C in Section 4.2 can be regarded as *i* and *j* respectively), we firstly deduce that the overlapping area between the communication area of *i* and *j* (denoted by $S_{ij}$) is proportional to the distance between *i* and *j* (denoted by $D_{ij}$). Next, we get the relationship between $D_{ij}$ and common neighbors of node *i* and *j* with the help of nodes’ uniform deployment (see Figure 3). As Figure 4 shows, $S_{ij}$ can be denoted by the equation below:

(1) $$S_{ij}=S_{AO_{i}B}+S_{AO_{j}B}-S_{AO_{i}BO_{j}},$$

where $S_{AO_{i}B}$ and $S_{AO_{i}B}$ represent the area of sector $AO_{i}B$ and $AO_{j}B$, respectively, and $S_{AO_{i}BO_{j}}$ denotes the area of rhombus $AO_{i}BO_{j}$. Then, based on geometry theory, we can easily get that

(2) $$S_{ij}=2R^{2}cos^{-1}\frac{D_{ij}}{2R}-D_{ij}\sqrt{R^{2}-\frac{D_{ij}^{2}}{4}},$$

where *R* is the communication radius of node.

**Figure 5:**
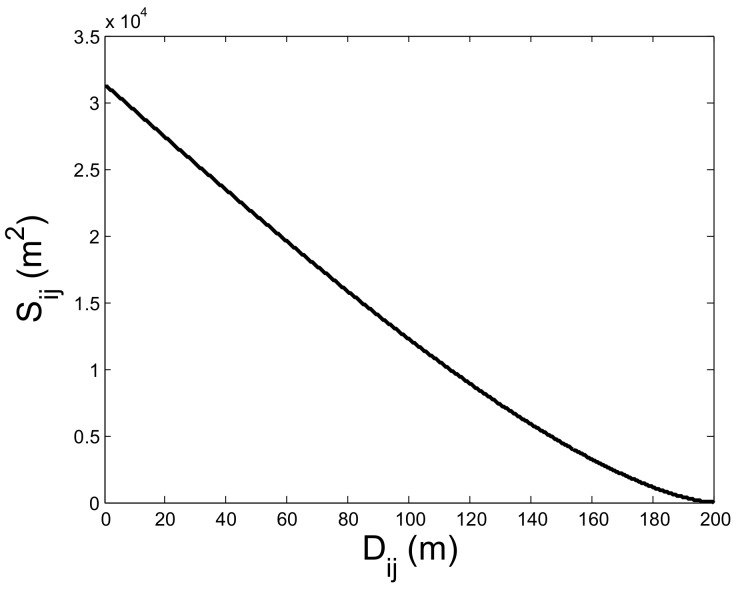
The relationship between $S_{ij}$ and $D_{ij}$.

From Equation (2), $S_{ij}$ monotonically decreases with the increase of $D_{ij}$. When $D_{ij}=R$, $S_{ij}=0.391\pi R^{2}$. It means that *i* and *j* may not be neighbors when $S_{ij}<0.391$ and they are likely neighbors when $S_{ij}\geq 0.391$. Thus, $S(D_{ij}=R)=0.391$ is regarded as a threshold value. Using Equation (2), we plot the relationship between $S_{ij}$ and $D_{ij}$ in Figure 5. Since $S_{ij}$ is approximately proportional to $D_{ij}$, Equation (2) can be simplified to Equation (3), where α is a constant:

(3) $$S_{ij}=\alpha D_{ij}.$$

Since nodes are uniformly deployed, the average area where only one node lies (denoted $S_{\varepsilon }$) is $\frac{\pi R^{2}}{N_{i}}$, where $N_{i}$ is the number of one node’s neighbors. Thus,

(4) $$S_{ij}=S_{\varepsilon }N_{c}=\frac{2\pi R^{2}N_{c}}{N_{i}+N_{j}},$$

where $\frac{N_{i}+N_{j}}{2}$ is the average value of the two nodes’ neighbors, and $N_{c}$ is the common neighbors of *i* and *j*. Combining Equations (3) and (4),

(5) $$D_{ij}=\frac{\beta N_{c}}{N_{i}+N_{j}},$$

where $\beta =\frac{2\pi R^{2}}{\alpha }$. Suppose that $D_{u}$ is the distance threshold value for whether the recommended node needs to be verified. When the receiving node detects the recommended node, it calculates the distance ratio *D*. When $D>D_{u}$, it indicates that the recommended node is close to the receiving node, and is likely a neighbor, hence is selected to be verified, and vice versa.

**Figure 6:**
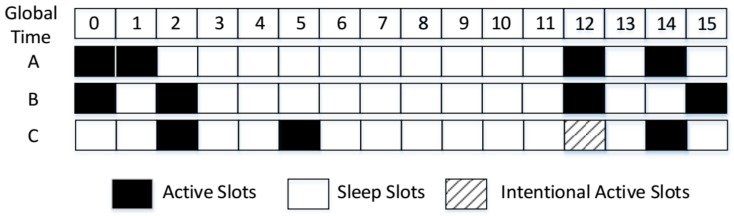
The benefit of early discovery.

Through the verification of recommended nodes stated above, GBFA achieves some early discoveries. However, there is one problem: it is not very energy efficient to verify the node whose early time may be much smaller. Take Figure 6 for example. A, B and C are three sensor nodes. They all employ a searchlight pairwise method and GBFA to achieve discovery. Their DCs are all $\frac{1}{12}$ in the Searchlight pairwise method. C discovers B and knows the wake-up schedule of A in slot 2. C wakes up to verify A at slot 12 and discovers A in advance. If only Searchlight is used, C and A discover each other in slot 14, which is two slots behind slot 12, and the time of successful discovery when both Searchlight and GBFA are used. The early time is two slots and is very small. Thus, this verification is unworthy.

**Figure 7:**
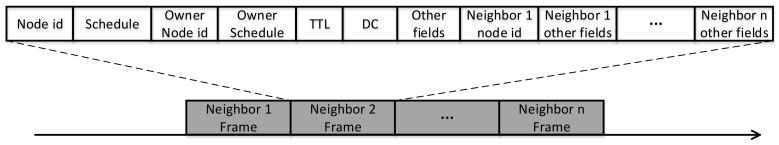
Beacon packet design.

We use gain ratio $P_{ij}$ to quantify the performance of each verification:

(6) $$P_{ij}=\Delta L_{ij}\sqrt{DC_{i}DC_{j}},$$

where $\Delta L_{ij}$ is the early discovery time of nodes *i* and *j* (the meaning of early discovery time is stated in Section 4.2), and $DC_{i}$, $DC_{j}$ represent the duty cycles of nodes *i*, *j*, respectively. $DC_{i}$ and $DC_{j}$ can be given before the network starts to work if all nodes work at the same duty cycle mode. Otherwise, duty cycle information is required to be carried in a beacon packet, as shown in Figure 7. $\sqrt{DC_{i}DC_{j}}$ is the geometric mean value of $DC_{i}$ and $DC_{j}$. We avoid that *P* is influenced by different DCs through multiplying the geometric mean value and $\Delta L_{ij}$.

We set a threshold $P_{u}$. If gain ratio $P\geq P_{u}$, this recommended node is selected to be verified. We can change the threshold based on the network environment and energy budget. When the energy budget is high, the value can be appropriately reduced to speed up neighbor discovery. Conversely, when the energy budget is low, the value is enlarged to save energy.

In summary, verification works if and only if $D>D_{u}$, $P>P_{u}$.

### 4.4. Slot Design

Different slot designs will greatly affect the discovery and transmission efficiency. If the time slot is shorter, there are more slots in one period, and the neighbor can be found more quickly. However, the shorter the time slot, the less information that can be transmitted within a time slot. In addition, when the time slot length is less than a value ( i.e., less than 5 ms), the timer jitter may cause serious problem [23]. Therefore, reasonable slot arrangements should be made according to different neighbor discovery algorithms and different application scenarios.

In LDC WSN, nodes are deployed and run independently, and there are often clock drifts between each node, resulting in only partial overlap between active slots of two nodes. To the best use of the overlap to achieve discovery, the Disco algorithm puts forward a kind of effective slot design. In both ends of one active slot, node sends a packet to broadcast its presence to other nodes. While the remaining time of the slot, the node is in a listening state to accept other beacons. To reduce the probability of conflicts caused by simultaneous sending, the listening time is usually several times longer than beacon time. Therefore, two nodes can still receive packets from each other in active slots and complete neighbor discovery, even in the case with large clock jitter.

Beacon is usually designed to be small in a normal discovery algorithm and can only send a Hello message to broadcast its existence, thus minimizing slot length achieves better performance. In the GBFA algorithm, to use the neighbor information to speed up the discovery process, it is necessary to carry additional neighbor information on the beacon packets. Thus, we have to expand slot length to send more information.

From Section 4.3, in order to select recommended nodes to verify, accepted nodes need to know the wake-up schedules of recommended nodes. In addition, the IDs of all neighbors of each neighbor are required to calculate a common neighbor number. When node density is large, neighbor number is too much, hence packet length increases. However, beacon length can’t unlimitedly grow. It is required to get appropriate packet size. In Disco, time slot length is 10 ms and each beacon probably only contains about 30 bytes of data. To carry the neighbor information in Acc [14], slot length is extended to 25 ms. Each beacon that can carry up to 80 bytes is enough to accommodate 40 IDs whose length is 2 bytes. In GBFA, we also set the default slot length of nodes to 25 ms, and we can make appropriate adjustments in different application scenarios and hardware platforms. When the transmission rate is higher or network node density is smaller, we decrease the length of time slot in order to obtain lower discovery delay. If sending rate is low or node density is larger, we can appropriately increase the length of slot in order to ensure that nodes can recommend enough neighbor information to each other.

If the packet length is too large in the network, it will occupy the communication channel for a long time. It is easy to cause collisions and the data transmission fails. In slot design of GBFA, our goal is: even if a small amount of neighbor information is lost, the algorithm can continue to use other neighbor information to speed up discovery. To achieve the goal, we can divide neighbor information into multiple frames, and the multiple frames are assembled into a beacon without gaps, so that the algorithm can continue to work even if some frames are lost. As shown in Figure 7, we show the arrangement of beacon information. The beacon is composed of multiple frames, each of which is used to store information of a neighbor: ID and work schedule of the node; ID, work schedule, TTL (Time To Live) and DC (duty cycle) of its owner node; and IDs and other necessary fields of neighbor nodes. As a result, the algorithm still can continue to work as long as at least one frame is received.

## 5. Evaluation and Performance Analysis

We use Java language to simulate the algorithms. In the simulations, all nodes are randomly deployed in a 500 m × 500 m two-dimensional rectangular monitoring area. The random node movement model [31] is used to simulate the movement of the sensor nodes. We compared GBFA with other two middleware algorithms, i.e., Group-based and Acc. In every scenario, two pairwise discovery algorithms Disco and Searchlight are employed in an underlying layer, respectively. To be fair, we made the total DCs (included the DC of pairwise method and the additional DC of middleware methods) of all middleware methods the same. Then, we compared their CDF (cumulative distribution functions) or average discovery latencies (for short, ADLs). We list the main evaluation parameters in Table 2.

### 5.1. CDF Comparison

We simulated three middleware algorithms in the static scenario and compared the changes of their CDFs with an increase of time slots. We adjusted the total DCs of all methods to 2%.

**Figure 8:**
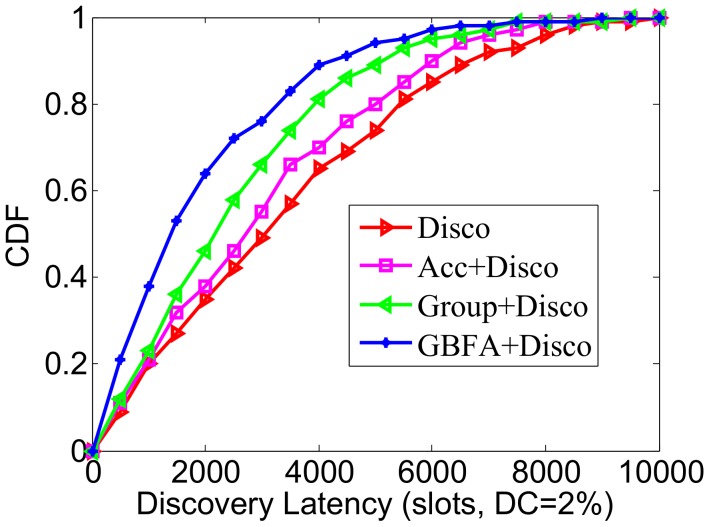
CDF comparison when the pairwise method is Disco.

Firstly, Disco is used as an underlying pairwise discovery algorithm. We can see from Figure 8, over time, that the algorithms discover all neighbors within their worst-case bound time. Disco finished all discoveries in 9854 slot. Acc, Group-based and GBFA respectively completed all discoveries in 9236, 9127 and 8818 time slots. In addition, before the 2000 slot, the change of the four methods is similar, and in 2000–4000 slots, the Group-based method’s convergence speed is quicker than Acc and Disco, and the convergent speed of GBFA is faster than the Group-based again. For example, in the 4000 slot, Disco found 65.2% of neighbors, Acc found 70.3% of neighbors, Group-based found 81.5% of neighbors, and GBFA found 88.7% of neighbors. This is because, at the beginning, almost all nodes haven’t found their neighbors, so that the indirect information used to neighbor discovery is less. However, with the passage of time, the number of neighbors to be recommended increases and the discovery process is accelerated accordingly.

**Figure 9:**
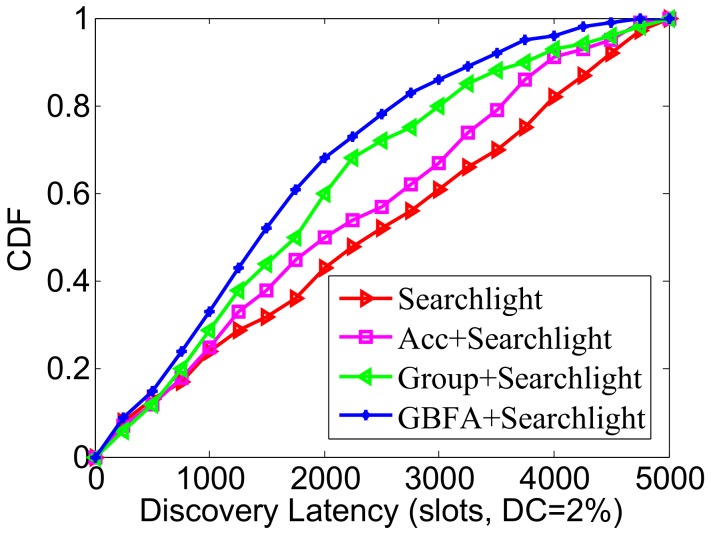
CDF comparison when the pairwise method is Searchlight.

Next, Searchlight is used as the underlying pairwise discovery algorithm. We compared the CDFs of Searchlight and other three middleware methods (see Figure 9). The worst-case latency bound of Searchlight is roughly half of the one of Disco. Thus, the CDF’s convergence speed of one middleware over Searchlight is much less than that when the pairwise method is Disco. For example, in 3000 slots, GBFA+Searchlight finds 86.3% of neighbors and GBFA+Disco only finds 68.1% of neighbors. In addition, GBFA+Searchlight always outperforms Group-based+Searchlight and Acc+Searchlight.

### 5.2. Impact of DC

By making DC change from 1% to 5%, we compare the ADLs influenced by DC.

**Figure 10:**
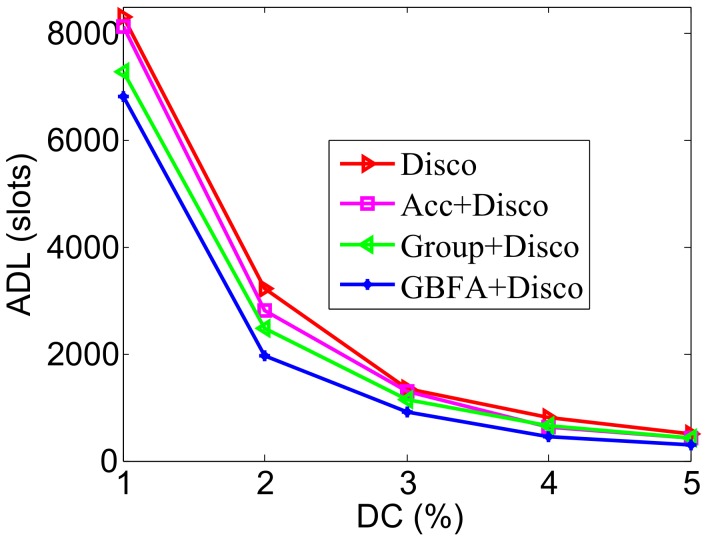
Impact of duty cycle when the pairwise method is Disco.

**Figure 11:**
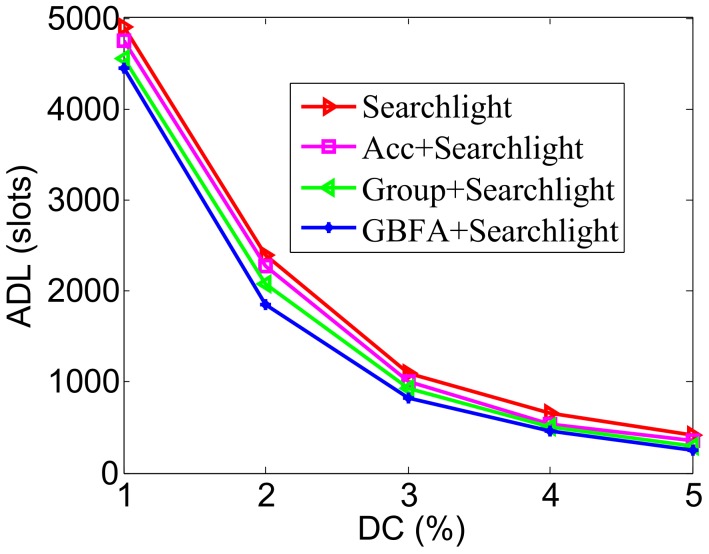
Impact of duty cycle when the pairwise method is Searchlight.

In Figure 10, the pairwise method is Disco. The ADL of GBFA is lower than Disco and the Group-based method. This effect is especially significant when the DC is low. For example, when the DC is 2%, ADLs of Disco are 3247, Acc’s ADL is 2800, the Group-based method’s ADL is 2240, and GBFA’s is 2003. Compared with Group-based, GBFA decreases the ADL approximately by 10.58%. The Group-based method requires additional active slots for transmitting recommendation information. GBFA may verify before the Group-based method’s transmission slot and discovered neighbors. The lower the DC, the more obvious the differences are. In Figure 11, the pairwise method is Searchlight. Compared with Disco-based cases, the ADLs of all middleware methods are much lower. Compared with other middleware methods, the ADL of GBFA+Searchlight is also lower. For example, when DC is 2%, the ADLs of Group-based+Searchlight and GBFA+Searchlight are 2078 and 1843 slots, respectively. GBFA+Searchlight decreases the ADL approximately by 11.31%, compared with Group-based+Searchlight.

### 5.3. Impact of Node Density

We placed 32 nodes in the initial network, adding 32 nodes each time, up to 704 nodes, that is, the node density is from 1 to 22. We compared the ADLs with the increase of node density when total DCs of all methods always remain 2%.

**Figure 12:**
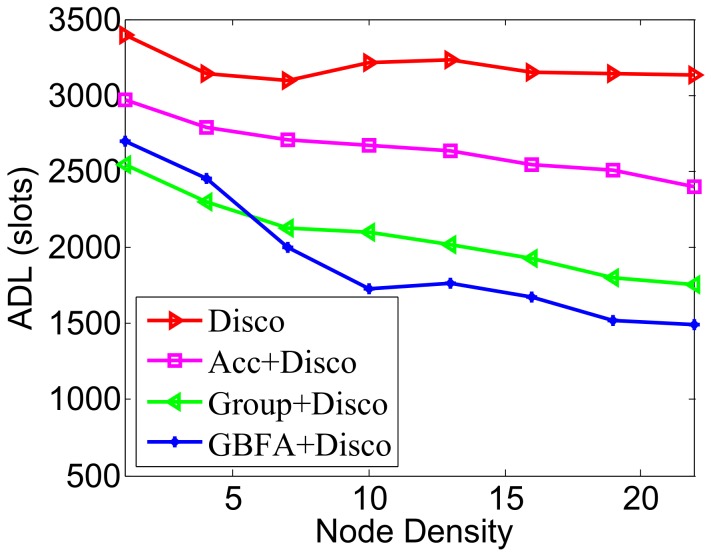
Impact of node density when the pairwise method is Disco.

**Figure 13:**
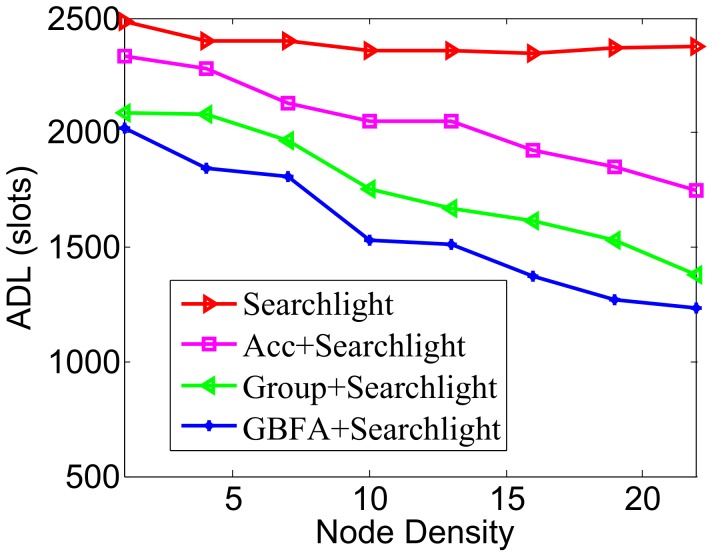
Impact of node density when the pairwise method is Searchlight.

In Figure 12, the pairwise method is Disco. The ADL of Disco remains almost unchanged when node density enlarges, while Acc, Group-based and GBFA decrease the ADL with the increase of node density. The reason is, as the node density enlarges, the more neighbors of the node are, the more neighbor information used to speed up discovery is. When node density is more than 8, the average discovery delay of GBFA has been lower than Group-based, which indicates that the performance of GBFA is better than Group-based when the node density is larger. When node density is not more than 8, Group-based’s average latency is lower than GBFA. It is likely because the effective recommendations’ number is smaller in GBFA in this case. In Figure 13, the pairwise method is Searchlight. The impacts of node density on ADLs of the three middleware methods are similar to Figure 12. When the node density is up to 22, the ADLs of Searchlight, Acc+Searchlight, Group-based+Searchlight and GBFA+Searchlight are 2378, 1750, 1378 and 1233 slots, respectively.

### 5.4. Impact of Speed

The moving speed of node starts from 5 m/s, and each time increases at a speed of 5 m/s, until the moving speed reaches 40 m/s. We compared ADLs of all methods.

**Figure 14:**
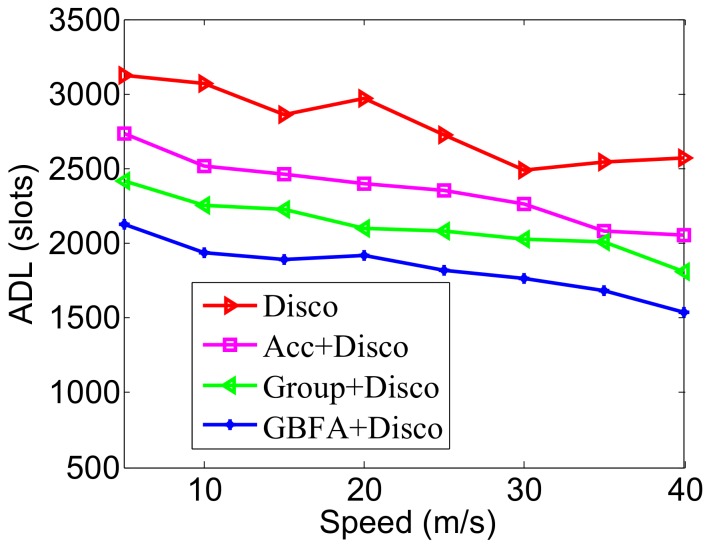
Impact of speed when the pairwise method is Disco.

**Figure 15:**
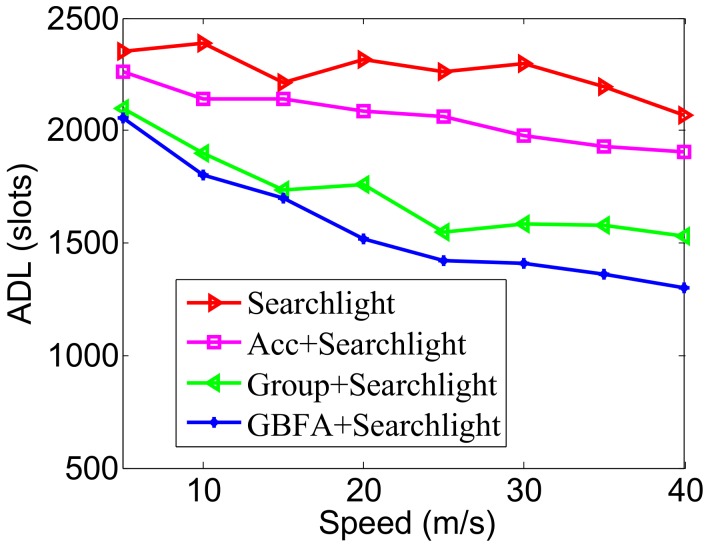
Impact of speed when the pairwise method is Searchlight.

In Figure 14, the pairwise method used is Disco. We find that, as the speed enlarges, the ADLs of the four algorithms decrease. This is because the larger the speed is, the shorter the average time from two nodes’ encounters to their departure is. Many nodes take a long time to discover each other but are too late to discover the others that have left the communication range of the nodes. In addition, we can see that Acc, Group-based and GBFA still achieve lower ADL; in particular, the ADL of GBFA is lower than the other three algorithms at various moving speeds. For example, when the node moves at a speed of 25 m/s, the ADL of GBFA is only 1817 slots, while Group-based, Acc and Disco are 2080, 2351, 2726 slots, respectively. In Figure 15, the pairwise method is Searchlight. The changes of ADLs with the increase of speed are similar to Figure 14. When the node speed is 40 m/s, the ADL of GBFA is only 1305 slots, but Group-based, Acc and Disco are 1528, 1902, 2070 slots, respectively.

## 6. Discussion

Our proposed method, i.e., GBFA, performs well in various cases and is especially suitable for IoT-oriented WSNs scenarios with high node density. As a universal networking technology, GBFA can be used not only in WSNs but is also easily extended to other wireless ad hoc networks, i.e., mobile phone based P2P networks. In such scenario, many people enter into the network area and lots of people leave the area at the same time. Since the network topology may change very frequently, a neighbor discovery method must work efficiently to be aware of the changes. The increase of discovery latencies will lead to packet loss, hence degenerates the performance of the upper application. For example, in a P2P social application, large latency discovery or unreliable discovery will lose some potential friends. GBFA can obtain outstanding advantages in the case.

Although GBFA performs well than other related methods, it still has some disadvantages. Firstly, its performance is not good when the node density is low as stated in Section 5. In the future, we will study how to propose a self-adaptive method based on node density. When the node density is low, only a pairwise method or another middleware program can be employed. When the node density is more than a threshold value, discovery middleware can be used. In the self-adaptive method, how to automatically measure the node density is very important and neighbor discovery rate can be used as the basis of quantitative analysis of node density. Secondly, only neighbor table information is used in GBFA and the information is insufficient for neighbor discovery in mobile WSNs. Other additional information, i.e., node motion model and the information about node location, can be utilized. For the process of leaving nodes, the TTL can be set according to the maximum speed of nodes. In the case of large differences in node speeds, sometimes, the TTL value is too short and the discovery performance is reduced. In the future, we will use the specific motion model to accurately estimate the neighbor’s hold time and set the corresponding TTL value. Thirdly, with the increase of node density, the size of the beacon will increase to carry more neighbors’ information, so that packet collisions increase. GBFA fails to quantify the relationship between node density and packet collision number. Based on this analysis, selecting more effective neighbor information and removing the others to reduce the beacon size may improve the energy efficiency of the method. This is our research direction in the future because of its complexity. Finally, the core idea of GBFA is to add a small amount of energy efficient active slots and this measure may not be the most energy efficient, particularly in dense WSNs. Adding active slots means the increase of the possibility of packet collisions. In the next part of our research, we will combine adding proactive active slots and removing unnecessary active slots whose scheduling rights are owned by underlying pairwise neighbor discovery method. It is worth stressing that adding or removing active slots is strongly interrelated to other factors, i.e., node motion model, except neighbor table information. In short, our goal in the future overall is to take the factors discussed above into consideration and find a neighbor discovery method with high performance to meet the requirements of rapid development IoT-oriented WSNs and other WSNs.

A main limitation of our work in the paper is that we fail to implement the system in a real-world scenario. In the future, we will verify GBFA though real-world experiments in industrial WSNs, P2P wireless social ad hoc networks or person health and safety field, i.e., using WSNs to monitor the physiological and biomechanical parameters of the athlete and bike, respectively.

## 7. Conclusions

With the rapid development of IoT, existing neighbor discovery methods in WSNs fail to satisfy some requirements in IoT-oriented WSNs. To address this issue, this paper proposes a group-based fast neighbor discovery algorithm GBFA in LDC WSNs, which filters out verifications with low efficiency as much as possible and hence improves the energy efficiency. The simulaions indicate that GBFA ourperforms outperforms other methods in various cases. Although GBFA performs well, it still has some limitations. In the future, our goal is to further improve the energy efficiency of neighbor discovery in WSNs, based on GBFA.

## Figures and Tables

**Table 1:** Related work summary.

Categories	Sub-Categories	Main Works
pairwise methods	probabilistic methods	Birthday [19], PSBA ${}^{1}$ [20], Panda [12]
pairwise methods	deterministic methods	Quorum-based [21,22,29]; Prime-based: Disco [23], U-connect ${}^{2}$ [24]; Searchlight [7]; Unequal-sized Slot methods [7,25,26]; Block-based [8,27];
middleware methods		WiFlock [13], Acc ${}^{3}$ [14], EQS ${}^{4}$ [9], Group [10]

${}^{1}$ prime-set-based algorithm; ${}^{2}$ unified connect; ${}^{3}$ on-demand accelerations; ${}^{4}$ extended quorum systems.

**Table 2:** Simulation parameters.

Parameters	Descriptions
network area size	500 m × 500 m
node communication radius	50 m
node number	default value: 200, variation range: [32, 704]
node movement speed	default value: 5m/s, variation range: [0, 40]
average DC	default value: 2%, variation range: [0.01, 0.05]
slot length	default value: 25 ms
